# Environmental exposures, microbiome dynamics and chronic disease risk in climate-vulnerable regions: interdisciplinary perspectives from Puerto Rico

**DOI:** 10.3389/fpubh.2026.1807779

**Published:** 2026-04-30

**Authors:** Filipa Godoy-Vitorino, Pablo Méndez-Lázaro, Benjamin Bolaños-Rosero, Abel Baerga-Ortiz, Polaris Torres-Rodríguez, María A. Sosa, Mariam Vázquez, Nydia L. Rivera-Rivera, Nancy Cardona-Cordero, Vivian Colón-López, Ana P. Ortiz

**Affiliations:** 1 Department of Microbiology and Immunology, School of Medicine, University of Puerto Rico, San Juan, PR, United States; 2 Puerto Rico Center for Microbiome Sciences, School of Medicine, University of Puerto Rico, San Juan, PR, United States; 3 Environmental Health Department, Graduate School of Public Health, University of Puerto Rico, San Juan, PR, United States; 4 Cancer Control and Population Sciences Program, Comprehensive Cancer Center, San Juan, PR, United States; 5 Department of Biochemistry, School of Medicine,University of Puerto Rico, San Juan, PR, United States; 6 Department of Anatomy and Neurobiology, School of Medicine, University of Puerto Rico, San Juan, PR, United States; 7 Department of Biostatistics and Epidemiology, Graduate School of Public Health, Medical Sciences Campus, University of Puerto Rico, San Juan, PR, United States

**Keywords:** environmental health, human microbiome, air pollution, chronic disease risk, climate vulnerability

## Abstract

Environmental factors such as air pollution, weather events, and ambient toxins are major contributors to human disease, with disproportionate impacts on vulnerable populations. In Puerto Rico, chronic exposure to air pollution and ecological disruption poses significant public health risks, particularly for cancer and other chronic conditions. These risks are unevenly distributed, disproportionately affecting children and older adults, groups central to community resilience yet highly susceptible to pollution-related health effects. This perspective review synthesizes emerging evidence linking chronic air pollution and environmental exposures to cancer, respiratory and cardiovascular disease, and microbiome alterations that may mediate long-term health trajectories. Drawing on interdisciplinary efforts from the Caribbean Cancer Research Center on Environmental and Natural Hazards, the Center for the Promotion of Cancer Health Equity, the Caribbean Collaborative Action Network, a NOAA CAP/RISA Team, and the Puerto Rico Center for Microbiome Sciences, this paper examines how environmental exposures shape health disparities. We highlight studies demonstrating that fungal spores, particulate matter, and chemical pollutants disrupt microbiome balance, immune regulation, and metabolic pathways, thereby increasing disease risk in early life and aging populations. The review also considers social determinants of health, spatial inequities, infrastructure vulnerabilities, and policy frameworks that influence exposure and resilience. By integrating environmental epidemiology, microbiome research, and public health policy, this synthesis underscores the urgency of planetary health–informed prevention, surveillance, and management strategies to mitigate pollution-related disease burdens, reduce inequities, and strengthen health in climate-sensitive regions globally.

## 1 Introduction

Environmental, biological, and social determinants converge to shape health outcomes in Puerto Rico (PR) and the broader Caribbean region. The goal of this perspective is to propose an interdisciplinary framework linking environmental pollution, climate-driven stressors, microbiome alterations, and downstream health outcomes. By synthesizing evidence across environmental epidemiology, microbiome science, and public health, we highlight how interacting ecological and social determinants shape disease risk and health inequities in climate-sensitive regions. Within this dynamic and vulnerable region, multiple stressors such as extreme weather events, species extinctions, urbanization, and air pollution interact to amplify health inequities and threaten the well-being of entire communities in small island states and territories (1). Their geographic isolation, concentrated exposure pathways, and limited infrastructure resilience make them particularly sensitive to environmental perturbations. As a U.S. territory with well-characterized environmental monitoring systems and research infrastructure, Puerto Rico offers a unique opportunity to integrate local environmental observations with global environmental health research. These phenomena are global, situating Puerto Rico within the broader literature on atmospheric pollution and environmental health (2). Against this backdrop, in Puerto Rico four complementary initiatives, the Puerto Rico Center for Microbiome Sciences (PR-CMS), the Center for the Promotion of Cancer Health Research (CePCHe), the Caribbean Cancer Research Center on Environmental and Natural Hazards (CARIB-CARES), and the Caribbean Collaborative Action Network (CCAN)-A NOAA CAP/RISA Team, represent a coordinated, science-driven response to address these intersecting scientific challenges. Together, these initiatives provide the scientific foundation to investigate how environmental exposures—including pollution, climate variability, and ecological disruption—interact with human and environmental microbiomes to influence chronic disease risk. They aim to strengthen research capacity, promote health equity, and develop adaptive strategies to enhance resilience across the life course. Puerto Rico represents a particularly informative setting for environmental health research due to the convergence of industrial contamination, climate vulnerability, and health disparities within a relatively small geographic area. Notably, the island has one of the highest densities of Environmental Protection Agency (EPA) Superfund sites per square mile in the United States, reflecting a legacy of industrial activity and hazardous waste disposal that continues to influence environmental exposures. By linking microbiome science, cancer health equity, and environmental hazard research, Puerto Rico is establishing a sustainable foundation for interdisciplinary discovery and regional leadership. This collective effort underscores the island’s growing role in shaping evidence-based strategies that address environmental determinants of health while empowering future generations of scientists and communities to thrive in the face of environmental change.

### 1.1 Environmental risks and pollution burdens in Puerto Rico

Puerto Rico faces a complex interplay of environmental risks that reflect its geographic vulnerability, industrial legacy, and socio-political context (3). The island’s unique topography and dense urbanization patterns amplify exposure to multiple pollutants, including fine particulate matter (PM₂.₅), which has been associated with risk of pre-term birth (4), volatile organic compounds, and toxic metals (5) released from vehicular and maritime traffic emissions and industrial waste. Given that many communities reside near petrochemical refineries, landfills, and former military sites that continue to leach toxic substances into soil and groundwater, compounding risks for chronic diseases (6). These structural inequities interact with environmental exposures, making Puerto Rico an important setting for examining how social determinants modify the health impacts of pollution and climate stressors. Air quality deterioration, exacerbated by aging infrastructure, unregulated emissions, and post-hurricane debris burning, has been linked to rising rates of respiratory illness, cardiovascular disease, and cancer. Beyond direct human health impacts, pollution also destabilizes surrounding ecosystems, accelerating biodiversity loss and disrupting natural barriers that once mitigated environmental hazards such as flooding and heat exposure. Social and economic inequities further shape patterns of vulnerability: low-income and rural populations, as well as children and older adults, are disproportionately affected due to limited access to healthcare, inadequate infrastructure, and historical disinvestment in environmental monitoring (7). These converging pressures underscore Puerto Rico’s urgent need for comprehensive strategies that integrate environmental health equity and sustainable development to protect both human and ecological resilience.

### 1.2 Fungal exposures, Saharan dust and respiratory health risks in Puerto Rico

The Caribbean region is affected by natural sources of air pollution such as aerosols (e.g., Saharan Dust) and aeroallergens (such as pollen, spores, and mold). Dust particles carried across the Atlantic from the Sahara and Sahel deserts, traveling thousands of kilometers (mostly during June–July–August), affect climate, weather, various ecosystems (including coral reefs, forests), and human populations (8–12). Saharan dust provides nutrients to terrestrial and marine ecosystems; however, in the Caribbean, Saharan dust has been associated with increased cardiovascular and respiratory risks and asthma in children and may cause cytotoxicity to the respiratory system (13–15). Since 2020, Puerto Rico has an Early Warning System (EWS) for Saharan Dust (https://aerosoles.caricoos.org/) providing near real-time information, risk levels, and forecasting information. This EWS is based upon work supported by the National Aeronautics and Space Administration (NASA), under Grant No. 80NSSC19K0194 issued through the Applied Sciences Program of the NASA Earth Science Division.

Fungi are ubiquitous eukaryotic organisms that play essential roles in both environmental and medical contexts (16). Exposure to airborne fungi is a well-recognized contributor to adverse human health outcomes, underscoring the need for routine environmental monitoring (17). Fungi reproduce by releasing spores, which are microscopic particles that range in size from approximately 0.65 μm to 45 μm. This size range affects their gravitational settling and their interactions with air turbulence. In tropical rain forest air, fungal particles can contribute up to 35% of PM_10_ mass (18). These exceptionally high fungal bioaerosol loads, driven by Puerto Rico’s tropical climate and frequent precipitation, create a unique setting to investigate how airborne microbial exposures interact with human microbiomes and respiratory health.

The health impact of fungal spores largely depends on their aerodynamic properties. Spores smaller than 2.5 μm can reach the alveoli in the lungs and trigger asthma in sensitized individuals (19). In contrast, larger spores or spore clusters tend to deposit in the upper respiratory tract, contributing to allergic rhinitis (20). Elevated levels of airborne fungal spores, especially high concentrations, have been strongly associated with inflammatory respiratory conditions, such as asthma and allergic rhinitis (21).

In Puerto Rico, the prevalence of asthma is notably higher than in the continental United States. Approximately 2 in 13 people in Puerto Rico have current asthma, which represents about 15% of the population. In comparison, about 1 in 13 people in the United States (approximately 7%–8%) have asthma (22). Due to its tropical climate and frequent precipitation, Puerto Rico experiences exceptionally high concentrations of airborne fungal spores, with spore counts often exceeding 110,000 spores/m^3^, more than twice the levels recorded at other National Allergy Bureau (NAB) monitoring stations (23). Outdoor spore concentrations typically peak between September and November, coinciding with the island’s rainy season (24).

The ambient fungal community is predominantly composed of basidiospores (65%). Common Basidiomycetes found in Puerto Rico include *Pleurotus ostreatus, Chlorophyllum molybdites, Coprinus disseminatus*, and *Ganoderma applanatum* (25). Ascomycetes are mainly represented by *Aspergillus* species such as *A. fumigatus, A. terreus, A. niger*, and *A. flavus*, which are frequent causes of aspergillosis, lung inflammation, and significant triggers of asthma and allergic rhinitis (21, 26, 27).

Most homes in Puerto Rico rely on natural ventilation, which allows outdoor spores to easily infiltrate indoor environments through open windows and doors. Interestingly, the 2017 fungal spore season was unusually low, likely due to widespread vegetation loss following Hurricane María. However, the 2018 spore season reached the highest levels ever recorded in 20 years of data collection at the San Juan monitoring site. This surge was probably driven by the accumulation of decaying organic debris after the hurricane, which provided abundant substrates for fungal growth and spore proliferation. Interestingly, the hurricane phenomenon led to an increase in indoor proliferation of filamentous fungi, including the aforementioned allergenic *Aspergillus* species (28, 29). These record levels of fungal spores may have contributed to dysbiosis in the nasal microbiome of infants born in the aftermath of Hurricane María, with 42% abundance of *Alternaria* species in the noses of babies born in 2018, compared to just 9% in those born in 2019 (30).

### 1.3 Climate extreme events and extinctions

Extreme environmental disturbances are accelerating the loss of biodiversity across multiple ecological and biological scales, threatening the stability of both natural ecosystems and human health. Rising global temperatures, ocean acidification, and altered precipitation patterns are driving widespread habitat degradation, species migration, and extinction events (31). Climate projections for the Northeastern Caribbean suggest a future characterized by reduced precipitation and heightened magnitude and frequency of intense cyclonic storms, extreme heat episodes, and rising mean sea levels (1, 32). As a result, marine ecosystems are experiencing mass bleaching (33) as sea surface temperatures exceed thermal thresholds, disrupting the symbiotic relationship between corals and their algal partners (34). This collapse reverberates throughout marine food webs, leading to cascading declines in dependent species such as sea urchins, fish, and invertebrates that maintain reef balance and nutrient cycling (35, 36). Similarly, warming oceans and terrestrial pollution are altering microbial communities essential for ecosystem resilience, reducing the functional diversity of soil and aquatic microbiomes that underpin carbon sequestration and nutrient regulation (37, 38). In recent decades, Caribbean coral reef ecosystems have experienced severe biodiversity losses driven by the accelerating impacts of natural and environmental hazards and the spread of marine diseases (34). Among the most devastating events was the mass mortality of the long-spined black sea urchin (*Diadema antillarum*) in the early 1980s, which resulted in the death of more than 90% of the population across the region (39). This collapse fundamentally altered reef dynamics, shifting ecosystems from coral-dominated to algal-dominated states and disrupting critical ecological functions such as grazing and nutrient cycling. Nearly four decades later, *D. antillarum* populations have shown minimal recovery, leaving many reefs ecologically degraded and less resilient to additional stressors. Alarmingly, a new wave of sea urchin die-offs was reported across the Caribbean, including Puerto Rico, in early 2022—signaling an ongoing vulnerability of reef organisms to emerging pathogens and warming ocean conditions. Together with widespread coral bleaching, increasing ocean acidification, and intensified storm activity, these recurrent events underscore the profound and compounding threats that climate-related stressors pose to marine biodiversity and the ecological integrity of Caribbean reef systems. Puerto Rican scientists found several microbial dysbiosis post die off associated with animals collected in 2022, especially those with signs of disease, lacking keystone taxa normally found in healthy *Diadema*, including *Photobacterium* and *Propionigenium* (40). The island’s exposure to recurrent extreme weather events—including hurricanes, heat waves, and flooding—further amplifies environmental health risks by mobilizing contaminants, damaging infrastructure, and altering microbial ecosystems.

The human microbiome, intimately linked to environmental microbial diversity, is also undergoing profound shifts due to changing environmental conditions, urbanization, and exposure to pollutants. These perturbations reduce microbial richness, with clear rural to urban extinctions (41, 42), and disrupt host–microbe interactions, with implications for immunity, metabolism, and disease susceptibility. Thus, biodiversity loss, from coral reefs to the human gut, represents an interconnected crisis in which extreme environmental disturbances not only erode the planet’s ecological integrity but also undermines the biological foundations of human resilience and health.

### 1.4 Puerto Rico as a model for understanding compounded environmental stressors and advancing public health solutions

Island communities such as Puerto Rico are exposed to severe weather, including tropical cyclones, heat waves, and droughts, with impacts that are compounded by limited supply chains, weak infrastructure, and governance systems burdened by layers of bureaucracy that often lead to inaction (32, 43). People of the U.S. Caribbean have also been tempered by centuries of adverse sociopolitical and environmental issues, which have forced them to cope with adversity (44).

The Caribbean Cancer Research Center on Environmental and Natural Hazards (CARIB-CARES) adds a critical environmental dimension to this collaborative network. Caribbean islands such as Puerto Rico and the U.S. Virgin Islands face compounded vulnerabilities arising from geographic isolation, aging infrastructure, and exposure to multiple concurrent hazards. Intensifying atmospheric conditions have increased the frequency and severity of hurricanes, heat waves, and flooding events, all of which disrupt healthcare systems, mobilize environmental toxins, and compromise water and air quality. CARIB-CARES uses the novel approach of human-centered design, integrating the community into the development of strategies, efforts, and outputs. The Center’s research is well equipped to epidemiologically (1) assess the risk of cancer in Puerto Rico and the U.S. Virgin Islands in relation to natural and environmental hazards and social factors, and (2) evaluate the understanding of extreme weather events and their effects on pollution and the quality of life among cancer survivors. In collaboration with a coalition of partners from both territories, they will plan and implement culturally appropriate approaches to effectively translate findings into action across policy-community levels. Additionally, CARIB-CARES is committed to creating a new generation of scientists and leaders in the interdisciplinary topic of environmental stressors across the cancer continuum.

The Caribbean Collaborative Action Network (CCAN) (https://ccan-upr.org/) A NOAA CAP/RISA Team (established in the U.S. Caribbean in 2021) is a multidisciplinary team comprised of universities, agencies, and non-governmental organizations located in the Caribbean region, dedicated to addressing climate extreme challenges by providing scientific and technical knowledge, assessing needs, and co-producing capacities, strategies, and climate adaptation actions based on collectively generated insights and locally rooted, realistic scenarios. CCAN is a knowledge-action network in the U.S. Caribbean (Puerto Rico and the Virgin Islands) that assesses needs, offers technical and scientific expertise, facilitates communication, and builds transregional connections and capacity among community, territorial, and regional networks for climate extremes in the Caribbean. The NOAA Climate Adaptation Partnerships Program (formerly known as the Regional Integrated Sciences and Assessments (RISA) Program) works in regions across the United States since the 1990s to address local needs by providing relevant scientific expertise and resources. These teams’ partner with multiple private and public sector organizations across their regions, including local government agencies such as emergency management departments, utilities, ports, public health, small and medium-sized businesses, rural town and city planners, regional councils of government, among others.

Environmental risk is not equally distributed (environmental inequality). Structural determinants such as urban planning, infrastructure, and housing can exacerbate existing vulnerabilities or build resilience. According to the World Health Organization (WHO), urban planning, risk governance, and resilience have become increasingly important pathways to promote and protect public health at the local level, especially in the context of escalating environmental conditions. Urban planning shapes land usage, density, and green space, all of which influence flood risk and the effects (45) of heat islands.

Extreme events, including heat waves and tropical storms in Puerto Rico, have provided evidence of the critical role of infrastructure. Water, energy, and transport systems are critical to preserve health. Specific post-disaster studies documented that poor infrastructure and lack of preparedness of the basic systems may have contributed to 2,975 excess deaths (46). Ortiz and colleagues documented stressors faced by cancer survivors and how treatment interruption due to problems with energy and transportation systems lowered cancer survival (47). Apart from human health, ecosystems also face increased challenges with extreme events. Subject to hurricane disturbance for millennia, natural ecosystems of Puerto Rico exhibit clear patterns of resistance (e.g., many tree species have little immediate storm-related mortality) and resilience (e.g., leaf litterfall and stream chemistry returned to pre-hurricane levels in as little as 5 years) (48).

### 1.5 Health impacts of chronic pollution exposure

In the U.S. and territories, geographical areas heavily contaminated with potential harm to human health and the environment can be included in the Environmental Protection Agency’s (EPA) National Priority List (NPL) and designated as Superfund sites. Of the over 1,500 historical sites in the NPL scattered throughout the U.S., Puerto Rico has been home to 26, the highest density of sites by square mile in the U.S. Padilla and colleagues documented the historical contamination of groundwater resources in the north coast karst aquifers of Puerto Rico in (5), linked to dispersion of chemical compounds from selected Superfund sites in more recent studies, and documented spatiotemporal pattern changes due to climate events (49). Meeker and colleagues have extensively evidenced the presence of chemical contaminants among Puerto Rican women living near superfund areas and their association with adverse pregnancy outcomes, including preterm birth. Preliminary geospatial work has highlighted a high incidence of certain pediatric cancers near these sites in Puerto Rico (50).

In addition to Superfund sites, Puerto Rico is home to over 100 facilities that report toxic emissions to the EPA Toxic Release Inventory (TRI). In 2025, Cardona and colleagues documented for the first time the industries that reported toxic emissions for 15 years (2006–2020) and how living in a municipality with at least one industry that reported to the TRI increased cancer risk by 7%, when compared with people who live in municipalities with industries that do not report to the TRI (3). These industrial emission patterns highlight Puerto Rico as a valuable natural laboratory for examining the long-term health effects of chronic environmental contamination in small island settings. In Puerto Rico, the COBRE Center for the Promotion of Cancer Health Equity (CePCHE) directly addresses the profound disparities in cancer outcomes between Puerto Rico and the U.S. mainland.

Environmental degradation and social inequities intersect to produce a higher burden of pollution-related cancers, exacerbated by limited access to care, economic instability, and the lingering effects of poorly structured health systems.

### 1.6 Microbiome shifts and disease risk

The Puerto Rico Center for Microbiome Sciences (PR-CMS) (https://cobremicrobiomepr.com/) functions as a pioneering hub for microbiome research and education, fostering interdisciplinary collaboration, analytical innovation, and the professional development of emerging scientists (51). As a regional knowledge-action network, the Center unites researchers, clinicians, and community stakeholders to evaluate environmental and health needs, provide technical expertise, and build capacity for climate adaptation at local, territorial, and regional scales. Puerto Rico is uniquely suited for integrative studies of environmental change and human health because an unusually wide variety of ecosystems and exposure contexts—costal and estuarine zones, urban corridors, agricultural valleys, karst landscapes, dry forest, and tropical rainforest—occur within short travel distances across a small island footprint, enabling efficient comparative and longitudinal sampling. Through its integrative approach, PR-CMS advances understanding of how microbial ecosystems respond to and mediate the effects of environmental change, from soil degradation and pollution to disruptions in human and ecological microbiomes, and seeks to translate these insights toward prevention and improved treatment outcomes. By investing in microbiome literacy, data infrastructure, and hands-on training, the Center is positioning Puerto Rico as a regional and global leader in microbiome science and environmental health innovation.

A growing body of evidence from international research, as well as in Puerto Rico, demonstrates that shifts in the human microbiome are strongly associated with disease susceptibility, progression, and health disparities. Work led by Godoy-Vitorino and collaborators has been central to uncovering these connections, particularly among Hispanic women. Recent findings show that specific cervicovaginal microbial signatures—including alterations in bacterial diversity, fungal communities, and functional gene pathways—are linked to high-risk HPV infection and cervical dysplasia, underscoring the microbiome’s critical role in cervical disease progression in Puerto Rican women (52–54).

Interestingly, a novel study on the oral cavity examined how *environmental* fungi interact with the oral “resident” mycobiota and how those dynamics relate to periodontal disease in Hispanic adults in Puerto Rico (55). The authors performed full-mouth periodontal exams and characterized the oral mycobiome using ITS2 sequencing. At the same time, they obtained daily outdoor fungal spore counts from an aerobiology monitoring station and linked those environmental data to each participant’s recruitment day. The oral fungal community was clearly polymicrobial: common genera included *Candida*, *Saccharomyces*, *Rigidoporus*, *Aspergillus*, and *Trametes*.

The most novel aspect of the paper is the ecological link between outdoor environmental fungi and the oral mycobiome. By matching daily atmospheric spore counts to oral samples, the authors showed that as environmental spore levels increased, both richness and overall diversity of oral fungi tended to decrease. This pattern suggests that inhaled spores are not just passively deposited; they compete with or displace resident oral fungi, potentially interfering with adhesion and biofilm formation by commensal yeasts like *Candida*. In other words, the outside air acts as a continuous source of “microbial competitors” that can reshape the oral ecosystem. The paper’s key contribution is to frame periodontal health within a broader ecological context: disease risk is influenced not only by endogenous microbes and host factors, but also by the constant influx of environmental fungi that interact with, and sometimes outcompete, resident flora. This is the first study to quantitatively couple real-time outdoor spore levels with oral mycobiome profiles, highlighting that environmental exposures may modulate dysbiosis, inflammation, and ultimately susceptibility to oral and periodontal disease (55).

Extreme weather events and pollution-related exposures in Puerto Rico (56) plausibly also contribute to a substantial and uneven burden of dementia, as well as neurological and psychiatric conditions, with disproportionate impacts in older adults and in children/adolescents, respectively. Large-scale island-wide assessments conducted after Hurricane María documented clinically meaningful levels of posttraumatic stress and depressive symptoms among public-school youth, underscoring how disaster-related exposures can translate into population-level neuropsychiatric risk during sensitive developmental windows (57). In parallel, post-disaster contexts in Puerto Rico have been associated with disruptions and stressors affecting adult mental health, including among groups with high vulnerability, such as those exposed to repeated emergencies, resource insecurity, or displacement (58–60). These neuropsychiatric stressors unfold against a backdrop of rapidly accelerated population aging, implying an expanding burden of cognitive impairment and dementia—conditions for which environmental and inflammatory pathways are increasingly recognized as relevant and potentially modifiable (60, 61). As populations age, dementia is increasing, especially in regions with limited healthcare resources. Climate change further worsens outcomes for people with cognitive impairment. Puerto Rico illustrates this intersection: it has an aging population, high chronic disease rates, and intensified hurricanes, heat waves, and power outages that endanger people with Alzheimer’s disease (62). Extreme heat and pollution may also accelerate cognitive decline. Alzheimer’s is the fourth leading cause of death in Puerto Rico. Research shows gut microbiome imbalances, particularly in APOE ε4 carriers, may contribute to inflammation and disease progression (63). Furthers studies are warranted to relate dementia and microbiome with climate-associated health risks.

These observations motivate targeted research in Puerto Rico on microbiome-mediated mechanisms along the microbiota-gut-brain axis, including the mycobiome, to identify biologically plausible pathways that could link climate and pollution-related exposures to neuroinflammation, neurodevelopmental outcomes, cognitive aging, and psychiatric symptomatology. Environmental stressors known to affect Puerto Rico—such as episodic particulate matter events (including Saharan dust intrusions), industrial/urban air pollution, flooding-associated indoor dampness and fungal proliferation, and post-hurricane shifts in chemical exposures—can perturb microbial ecosystems and host immune signaling, thereby influencing brain-relevant pathways (immune, metabolic, endocrine, and vagal) (28, 56, 64–68). As a strategic priority, besides studies of cancer, metabolic and chronic diseases, the PR-CMS can also support longitudinal studies that integrate environmental measurements, along with bacterial and fungal multi-omics associated with dementia as well as measurements of neurobehavioral outcomes, accelerating the identification of actionable biomarkers and intervention targets to reduce neurological and psychiatric disease risk in Puerto Rico.

Collectively, these contributions position Puerto Rico as a critical site for understanding how social, environmental, and biological determinants shape microbiome variation and disease risk. Through ongoing projects, regional leadership efforts, and participation in global microbiome networks Puerto Rico microbiome research group continues to advance the scientific evidence base demonstrating that microbiome shifts are fundamental drivers of health outcomes across diverse contexts.

## 2 Conclusion

### 2.1 What’s next? Toward integrated prevention and health management

This perspective advances an interdisciplinary framework that integrates environmental exposures, climate dynamics, microbiome ecology, and health outcomes to better understand disease risk in climate-sensitive regions. As Puerto Rico confronts rising temperatures, degraded air quality as per its fungal load, Saharan dust intrusions, and increased fungal aeroallergen loads, fungi are emerging as a critical axis linking environmental change and human health. Extreme heat and humidity accelerate fungal growth in soils, decaying vegetation, and built environments, amplifying atmospheric spore concentrations during the island’s rainy season. Saharan dust episodes layer additional stress: dust plumes carry mineral particles, bacteria, and fungal propagules that can influence airborne microbiota and seed fungal growth. These exposures do not occur in isolation; Puerto Rico’s warming climate, urban heat islands, and moisture-damaged structures after recurring hurricanes create ideal conditions for fungal proliferation. The oral mycobiome study from Puerto Rico demonstrates how these environmental signals penetrate biological systems: as outdoor spore levels increase, resident oral fungal diversity decreases, reflecting direct ecological competition between environmental basidiospores and the mucosal fungi that normally anchor oral microbial homeostasis. This competition, where stress-tolerant environmental fungi displace or suppress commensal taxa like *Candida*, is likely to intensify under climate-driven heat events, when fungal sporulation surges. Integrating these insights is essential for developing intervention strategies that recognize fungi not merely as allergens or pathogens, but as dynamic environmental sentinels responsive to heat, pollution, and climate variability.

Emerging evidence from Puerto Rico underscores the need for microbiome-informed approaches to pollution mitigation that consider how fungal communities shift in response to atmospheric conditions, chemical exposures, and human behaviors. Heat stress and particulate pollution from vehicular emissions, industrial waste, and post-hurricane debris burning alter fungal physiology and may enhance the dispersal of stress-resilient taxa capable of colonizing human mucosa. This aligns with findings from oral mycobiome work in Puerto Rico: environmental fungi do not merely enter the oral cavity passively; they competitively reshape the mucosal ecosystem, influencing inflammation and disease susceptibility. Integrating these dynamics into environmental health frameworks opens new possibilities for early-warning systems that use fungal fluctuations as indicators of ecological stress, building on Puerto Rico’s existing Saharan dust early-warning system. Microbiome studies with a focus on mycobiota, human behavior, lifestyle, and climate should be a priority.

In clinical settings, preventive strategies for children who exhibit heightened asthma risks during high-spore seasons and aging adults, whose mucosal immunity declines with age, should incorporate fungal monitoring alongside bacterial microbiome analyses. Practical approaches include improving indoor air quality in schools and senior care facilities, deploying HEPA and dehumidification systems during high-spore periods, and crafting microbiome-supportive nutritional guidelines that maintain the resilience of the human mycobiota under environmental pressure.

These insights reinforce the need for interdisciplinary collaboration bridging environmental epidemiology, molecular microbiology, biomedical, health, and climate sciences, and public policy. Puerto Rico’s unique convergence of its wide variety of habitats and ecosystems with pollution burdens, climate hazards, and high fungal exposure provides an unparalleled natural laboratory to investigate how environmental fungi mediate the health impacts of heat stress and pollution. Moving forward, research priorities should include: (1) longitudinal cohort studies that couple fungal exposures with bacterial and fungal microbiome dynamics, immune markers, and chronic disease trajectories; (2) community-based monitoring systems that gather real-time fungal and pollution data to inform local adaptation strategies; (3) mechanistic studies to understand how heat-stressed fungi alter pathogenicity, allergenicity, or competitive fitness within the human microbiome; and (4) policy frameworks that integrate fungal ecology into climate resilience planning, housing standards, occupational health protections, and disaster-recovery protocols. Taken together, these efforts represent a call to action: to treat fungi as key environmental indicators, competitive ecological actors, and essential components of integrated health management. By embedding fungal ecology into environmental and public health strategies, Puerto Rico can lead the way in designing adaptive, microbiome-centered prevention programs that protect vulnerable populations across the life course.

We propose an integrative framework in which chronic exposure to environmental pollution, climate-driven increases in aeroallergen load and seasonality, and ongoing biodiversity loss act synergistically to alter both environmental and human-associated microbiomes. These disruptions reshape microbial community composition, diversity, and functional capacity across air, soil, water, and the human respiratory, gut, and skin ecosystems, mediating persistent inflammatory responses, impaired immune development and regulation, and heightened susceptibility to allergic, metabolic, cardiovascular, neurological, and autoimmune diseases. These effects are disproportionately borne by vulnerable populations, thereby exacerbating existing health inequities and contributing to long-term population-level disease risk. We further acknowledge the opportunity for multiple federally funded research centers to jointly leverage resources and expertise by establishing collaborative funding mechanisms, such as co-funded feasibility or pilot projects, focused on the intersecting domains of chronic environmental exposures, extreme weather events, immunology, and human microbiomes to accelerate interdisciplinary discovery and translation.
